# A Novel Technique for Microsurgery on Calcified Arteries: Venous Interposition Grafting

**Published:** 2019-07-02

**Authors:** Ray Christopher Hosein, Andrei Odobescu, Isak A. Goodwin

**Affiliations:** ^a^Division of Plastic & Reconstructive Surgery, University of Utah Health, Salt Lake City, UT; ^b^Division of Plastic & Reconstructive Surgery, University of Iowa Hospitals and Clinics, Iowa City, IA

**Keywords:** microsurgery, anastomosis, free tissue transfer, calcified vessels, limb salvage

**Figure:**
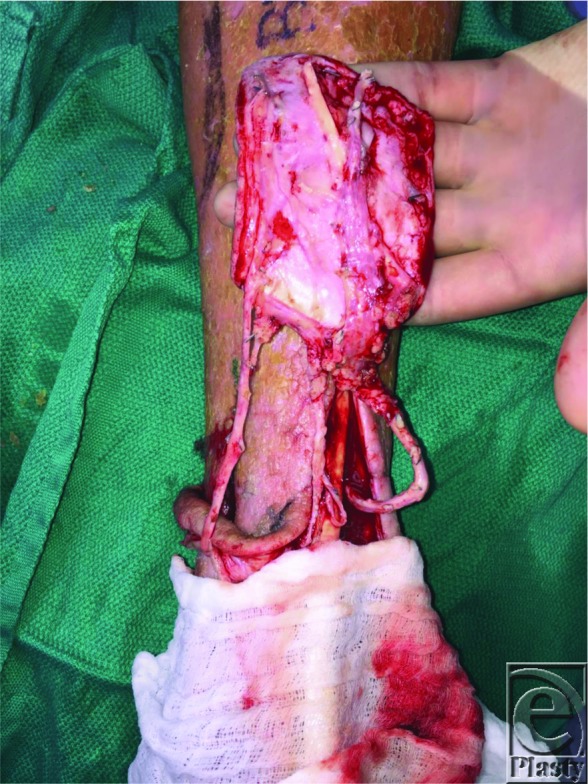
A Novel Technique for Microsurgery on Calcified Arteries: Venous Interposition Grafting The technique of venous interposition grafting is described in order to increase the success of micro-anastomoses involving severely calcified vessels.

The indications for free tissue transfer have expanded since the advent of microsurgery over the last 50 years.^1^ However, microsurgery on vessels containing calcified plaque presents several unique technical challenges. As calcified vessels thicken, they become stiff and difficult to handle during surgery. Needle and anastomotic holes may remain patent, leading to leaks as elasticity is lost. Plaque formation may lead to intimal delamination by the action of the needle being driven through the vessel wall.^2^ Several simple solutions can facilitate success when performing micro-anastomoses between 2 calcified vessels.

## INTIMAL DELAMINATION

The intimal and medial layers of the arterial wall can become calcified and inelastic with advanced age when loss of elastin and plaque deposition are abetted by hyperlipidemia, diabetes, and renal insufficiency.^3^ The fragile intima of vasculopathic vessels easily delaminates. Intimal delamination can be addressed by passing a needle from the luminal side to the adventitial side of the artery, effectively tacking the intima to the surrounding media and adventitia in the wall of the artery.^1^ Microsurgical suture is generally single armed, making it necessary to pass the suture from adventitia to the lumen on one vessel end and subsequently from the lumen to adventitia on the other vessel being anastomosed. The first half of this process can push the intima away from the media, creating an area of delamination, and potential nidus for thrombosis.

Interposition vein grafting for calcified vessel anastomoses can optimize the problem of intimal delamination by suturing from tunica externa to the lumen of the vein graft and the lumen to adventitia of the calcified artery. The same process can be undertaken at the other end of the graft, tacking the arterial intima up and preventing delamination.

## STIFF VESSEL HANDLING CHARACTERISTICS

Fragmentation of elastin and calcium deposits in vessel walls can also lead to poor handling quality when mobilizing vessels for anastomosis. Acute and chronic edema can further thicken vessel walls and encase the vascular tree in dense fibrotic tissue. These factors can both limit the available length of vessel that can be safely exposed and decrease the mobility of the vessel that is dissected free.^1^ Stiff handling characteristics make it more difficult to visualize the lumen after one or two sutures have been placed. Limited mobility can make the ergonomics of the anastomosis more challenging.

Anastomosing two calcified vessels directly together can place two noncompliant surfaces in contact, creating a poor seal and subsequent leakage. Commonly used anticoagulants, such as aspirin and heparin, may exacerbate hematoma formation in this patient population.^4^^,^^5^

Interposition vein grafting can add flexibility to the anastomosis that serves to improve the ergonomics and visualization of the intima.^1^ Because veins are less affected by calcification than arteries, the supple vein can be more easily sewn and allows one end of the anastomosis to be performed with improved degrees of freedom, followed by the second end of the anastomosis with equally improved handling and visualization.

## LEAKING SUTURE HOLES

Calcified plaque is difficult to puncture with standard microsurgery needles (eg, BV 130-5) and dulls the needle tip quickly. Use of cutting needles can lead to micro-tearing and leaks when sewing these delicate vessels. Leaking at suture holes can be difficult to solve, as further suture placement can lead to more leaking. Tapered needles with a cutting tip may help puncture the plaque while avoiding propagation of the puncture into a laceration (eg, Sharpoint, Wyomissing, PA).

## METHODOLOGY/CASE PRESENTATION

**Figure 1:**
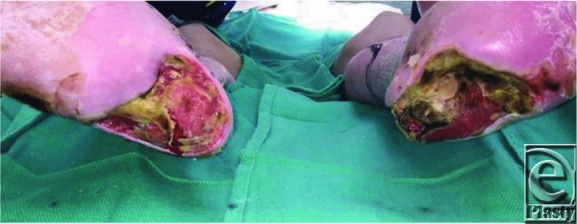
Bilateral chronic heel pressure ulcers with calcaneal osteomyelitis.

**Figure 2:**
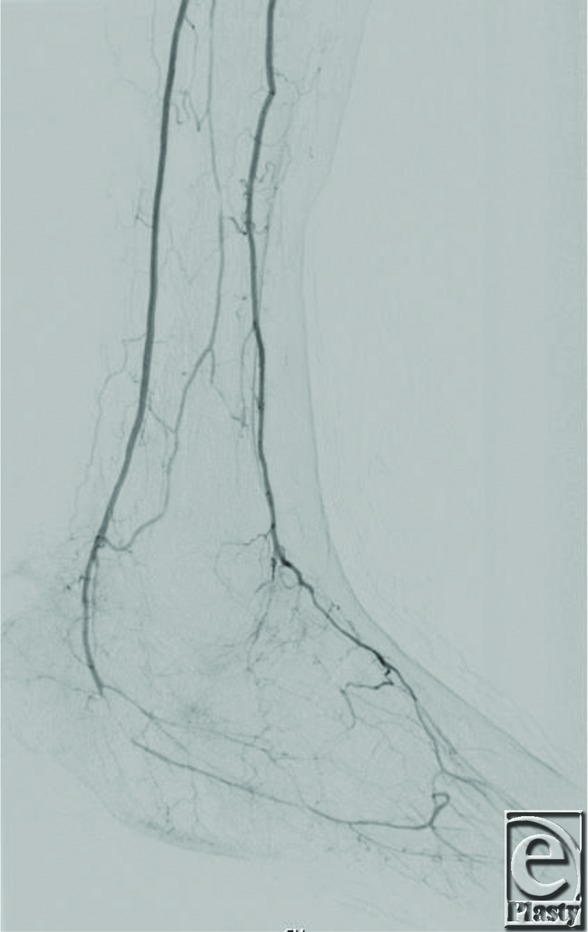
Right lower extremity angiography following angioplasty of the posterior tibial artery demonstrating restored inflow.

**Figure 3:**
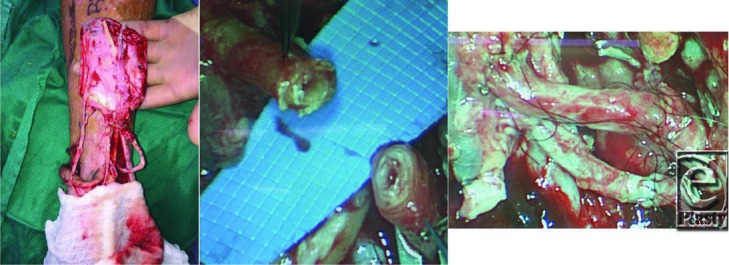
(a) Left dorsalis pedis spare part flap elevated on the anterior tibial artery pedicle and the greater saphenous vein pedicle. (b) Severely calcified and friable anterior tibial artery of the flap and the posterior tibial artery recipient pedicle. (c) 2.5-cm long interposition vein graft for arterial reconstruction, facilitating tension-free anastomosis with improved visualization and endothelial management.

**Figure 4:**
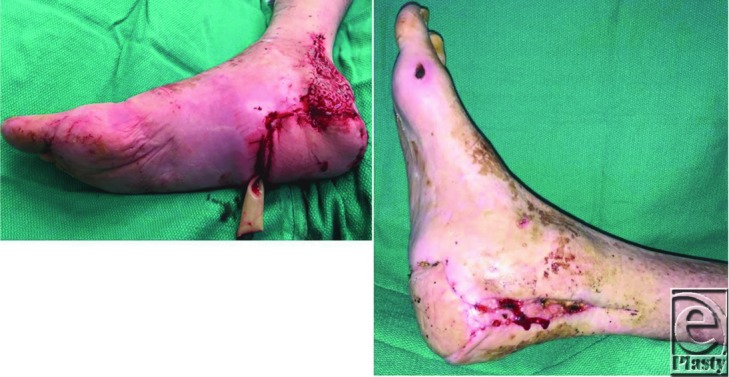
(a) Right heel soft tissue reconstruction with the dorsalis pedis spare part flap from the left foot. (b) Right foot at 6 weeks after surgery.

A 64-year-old previously ambulatory diabetic man with a history of tobacco use presented with bilateral stage IV pressure ulcers of the heels after an incapacitating illness (Fig 1). The wounds were grossly debrided, and bone biopsy samples were taken to guide antibiotic management for osteomyelitis. Ankle brachial indices were less than 0.4 for both extremities, indicating critical limb ischemia. Angiography showed multilevel arterial disease for the left lower extremity and a short-segment vascular occlusion on the right that was amenable to and subsequently underwent successful balloon angioplasty (Fig 2). The operative plan devised was to perform a left below-knee amputation and use spare parts from the amputated left leg in the form of a dorsalis pedis free flap to cover the right heel (Fig 3*a*). Both the donor and recipient arteries were significantly calcified, with delaminating intimal plaque and stiff, edematous walls (Fig 3*b*). Initial end-to-end anastomosis between the flap's anterior tibial artery and the recipient right posterior tibial artery was challenging due to poor mobility of the fibrotic recipient artery and intimal delamination. There was also significant leaking from the anastomotic edge and suture holes. To solve these problems, an interposition vein graft was used from the left greater saphenous vein, creating a more favorable anastomosis. This was particularly helpful at the stiff recipient right posterior tibial artery, which was anastomosed first. To secure the plaque to the underlying arterial wall, all sutures were placed from tunica externa to the lumen on the vein and the lumen to adventitia on the recipient posterior tibial artery as well as for the free flap's anterior tibial artery (Fig 3*c*). The final flap position after inset and anastomosis is shown in Figure 4*a*.

## RESULTS

Successful wound coverage was achieved at 6 weeks after surgery (Fig 4*b*).

## DISCUSSION

Heel ulcers in patients with chronic vascular disease often herald amputations due to poor reconstructive outcomes.^6^ Microsurgical options in situations of severe arterial calcification are often complicated by fibrotic peripheral vasculature with anastomotic leakage and intimal delamination.

Challenges related to the management of advanced peripheral arterial disease have become more prevalent in the Western society as the incidence of dysmetabolic disease rises.^3^ While the experience in vascular surgery for macrovascular disease in atherosclerotic vessels continues to advance for reestablishing inflow, that for surgery on microvascular diseased vessels is rarely reported in the literature.^1^^,^^6^^,^^7^ In addition, prior reports have described vein grafting in microsurgical anastomosis to solve the problem of inadequate pedicle length rather than to facilitate the anastomosis between calcified vessels.^1^^,^^8^ Mücke and others^9^ studied the efficacy of performing atherectomy in microvessels in a rat injury model and concluded that the intimal injury produced leads to a high risk of thrombosis after completing the anastomosis. Although this procedure may provide a more local solution to dealing with calcified macrovasculature, atherectomy is not usually a viable option for optimizing calcified vessels before micro-anastomosis due to its thrombogenic potential.

Finally, while venous interposition is a useful and versatile technique in difficult reconstructive situations, it can in itself be associated with increased rates of pedicle thrombosis due to flow passing through two anastomoses for each vessel.^10^ This limitation must be considered before resorting to this surgical option and should only be used when traditional anastomotic techniques either have failed or are likely to perform poorly.

## SUMMARY AND IMPLICATIONS

Interposition vein grafting can improve the ergonomics of microsurgical anastomosis in calcified vessels. The compliant nature of vein grafts can create an improved seal when anastomosed to the more rigid calcified artery. It is our hope that this simple technique could provide a reconstructable solution for this difficult clinical problem.
